# T Helper 1–Inducing Adjuvant Protects against Experimental Paracoccidioidomycosis

**DOI:** 10.1371/journal.pntd.0000183

**Published:** 2008-03-12

**Authors:** Leandro Licursi de Oliveira, Kely Cristine Coltri, Cristina Ribeiro Barros Cardoso, Maria-Cristina Roque-Barreira, Ademilson Panunto-Castelo

**Affiliations:** 1 Department of Cellular and Molecular Biology, School of Medicine of Ribeirão Preto, University of São Paulo, Ribeirão Preto, São Paulo, Brazil; 2 Department of Biochemistry and Immunology, School of Medicine of Ribeirão Preto, University of São Paulo, Ribeirão Preto, São Paulo, Brazil; 3 Department of General and Specialized Nursing, School of Nursing of Ribeirão Preto, University of São Paulo, Ribeirão Preto, São Paulo, Brazil; Hospital Universitário, Brazil

## Abstract

Immunostimulatory therapy is a promising approach to improving the treatment of systemic fungal infections such as paracoccidioidomycosis (PCM), whose drug therapy is usually prolonged and associated with toxic side effects and relapses. The current study was undertaken to determine if the injection of a T helper (Th) 1–stimulating adjuvant in *P. brasiliensis*–infected mice could have a beneficial effect on the course of experimental PCM. For this purpose, mice were infected and treated with complete Freund's adjuvant (CFA), a well-established Th1 experimental inductor, or incomplete Freund's adjuvant (IFA - control group) on day 20 postinfection. Four weeks after treatment, the CFA-treated mice presented a mild infection in the lungs characterized by absence of epithelioid cell granulomas and yeast cells, whereas the control mice presented multiple sites of focal epithelioid granulomas with lymphomonocytic halos circumscribing a high number of viable and nonviable yeast cells. In addition, CFA administration induced a 2.4 log reduction (>99%) in the fungal burden when compared to the control group, and led to an improvement of immune response, reversing the immunosuppression observed in the control group. The immunotherapy with Th1-inducing adjuvant, approved to be used in humans, might be a valuable tool in the treatment of PCM and potentially useful to improve the clinical cure rate in humans.

## Author Summary

*P. brasiliensis* is a thermally dimorphic human pathogenic fungus that causes paracoccidioidomycosis (PCM), the most prevalent human systemic mycosis in Latin America, whose drug therapy is usually prolonged and associated with toxic side effects and relapses. Although immunostimulatory therapy is a promising approach to improving the treatment of fungal infections as PCM, few studies have been reported. In the current study, we verified that a single-dose administration of an adjuvant that induces T helper (Th) 1 immune response (complete Freund's adjuvant [CFA]) in *P. brasiliensis*–infected mice was sufficient to break the lack of immune response to the fungus observed in infected mice. Four weeks after treatment, the CFA-treated mice presented a mild infection in the lungs characterized by preserved lung structure and small fungal burden, whereas control mice that had been treated with incomplete Freund's adjuvant presented many granulomatous lesions and high fungal burden. The immunotherapy with Th1-inducing adjuvant might be a valuable tool in the treatment of PCM and potentially useful for faster and efficient cure of PCM in humans.

## Introduction

*Paracoccidioides brasiliensis* is a thermally dimorphic human pathogenic fungus that causes paracoccidioidomycosis (PCM), the most prevalent human systemic mycosis in Latin America, being endemic in Brazil, Argentina, Venezuela and Colombia. This infection is acquired by inhalation of airborne propagules found in nature, which reach the lungs and are converted to the yeast form [1],[2]. The yeasts can either be eliminated by immune-competent cells or disseminated into tissues through lymphatic or hematogenous routes. PCM is characterized by granulomatous inflammation, intense immunological involvement with suppression of cellular immunity and high levels of non-protective antibodies in serum [3]. The disease may present a broad spectrum of clinical and pathological manifestations ranging from asymptomatic pulmonary infection to severe and disseminated forms [4],[5]. The chronic progressive form of the disease (CF) is the most common clinical presentation and predominantly affects adult males, with frequent pulmonary, mucosal, cutaneous and adrenal involvement. Although the outcome of the infection can be due to several factors, it is especially dependent on the protective capacity of the host immune system. The cell-mediated immune response represents the main mechanism of defense in PCM [1]. Conversely, it has been reported that a high level of humoral immune response is associated with increased disease dissemination [6].

The mechanisms underlying resistance or susceptibility to PCM remain to be elucidated. The development of the appropriate CD4^+^ T helper (Th) subset is important for PCM resolution and several studies have shown that different disease outcomes can be derived from the commitment of precursors to either Th1 or Th2 lineage [7],[8]. Resistance to *P. brasiliensis* infection has been related to interferon-γ (IFN-γ) and other Th1-type cytokines [9]–[11], while susceptibility has been linked to the preferential production of the Th2-type cytokines, i.e., interleukin (IL)-4, IL-5, and IL-10 [12]–[14]. Several investigators have suggested that progressive disseminated forms of PCM in humans are associated with various degrees of suppressed cell-mediated immunity [1],[15],[16]. This anergy can be reversed after successful therapy, when normal levels of T cell function are partially or completely restored [17].

The prognosis of PCM has been improved through antimycotic drugs, however treatment regimens require an extended period of time often associated with relapses. *P. brasiliensis* has the peculiarity of responding to treatment with sulpha drugs. Nevertheless, regimens with these agents often require extended period of maintenance therapy that may range from months to years. Clinically, the antifungal drugs most commonly used for PCM include amphotericin B, sulpha derivatives and azoles, but their toxicity can be a limiting factor in treatment [18],[19]. These concerns, together with the elucidation of the protective immune response against PCM have renewed interest in the development of alternative therapeutic strategies such as immunotherapeutic procedures, which can be useful for controlling PCM. The present study was designed to verify if immunomodulation with CFA could play a protective role in experimental PCM leading to a less severe infection with decreased fungal burdens in the lungs.

## Materials and Methods

### Fungal isolate

Yeast cells of virulent Pb 18 strain of *P. brasiliensis* were cultured at 37°C in YPD (Yeast Extract/Peptone/Dextrose) Medium (Difco Laboratories, Detroit, USA) for 7 days and washed three times in 0.01 M phosphate-buffered saline (PBS), pH 7·2. Viability of yeast cells was determined by the fluorescein diacetate-ethidium bromide treatment [20].

### Mouse infection and treatment

BALB/c mice, aged 6–8 wk, were bred and maintained under standard conditions in the animal house of the Medical School of Ribeirão Preto, University of São Paulo, Ribeirão Preto, SP, Brazil. All animal experiments were performed in accordance with protocols approved by the School of Medicine of Ribeirão Preto Institutional Animal Care and Use Committee. Mice were inoculated intravenously with 1×10^6^ viable yeast cells in 100 µl of PBS. On day 20 postinfection, mice were injected subcutaneously with 100 µl of CFA or IFA (Sigma Chemical Co., St. Louis, USA), both emulsified in PBS in a ratio of 1∶1. Mice were killed on day 30 after treatment and their lungs were aseptically removed. One lung from each mouse was used for histopathology analyses and the other for quantification of fungal burden and cytokines.

### Histopathology

The lungs were fixed in 10% neutral buffered formalin for 24 hours and embedded in paraffin. Tissue sections (5 µm) were stained with hematoxylin and eosin (H&E) or silver methenamine (Grocott) to detect the mycotic structures using standard protocols. Samples were analyzed by light microscopy in an Axiophot photomicroscope (Carl Zeiss, Jena, Germany) coupled with a JVC TK-1270 camera (Victor Company of Japan Ltd, Tokyo, Japan). The area of individual granulomas, as well as the total area of the lung sections and the area taken by granulomas per slide, was measured by computer-aided image analysis (ImageJ 1.37v, National Institutes of Health, Bethesda, USA). The following data were thus generated: granuloma area (mean area of all granulomas in each lung section), granuloma relative area (% represented by total granuloma area/total area of the lung sections) and number of granuloma cells per area (total number of cells from a granuloma section/the area of the respective granuloma section) of each mouse.

### Quantification of colony-forming units (CFU) and cytokines

The lungs were weighed and homogenized in 1 ml of sterile PBS using tissue homogenizer (Ultra-Turrax T25 Basic, IKA Works, Inc., Wilmington, USA). To determine the number of CFU, lung homogenates were diluted 1∶10 in PBS. Aliquots of 100 µl of each sample were dispensed into Petri dishes containing brain heart infusion agar (BHI, Difco) supplemented with 4% (v/v) of heat-inactivated fetal calf serum (FCS, Gibco BRL, Gaithersburg, USA). The plates were incubated at 37°C, the colonies were counted 14 days later, and then, the number of CFU per gram of tissue was calculated. For cytokine determination, remaining lung homogenates were centrifuged at 5,000×*g* for 10 minutes and the supernatants stored at −20°C until cytokine determination. Supernatants were analyzed as duplicate samples from replicate wells. A sandwich-type ELISA was used to determine IL-12, IFN-γ, TNF-α, IL-4, IL-10, and TGF-β levels, using OptEIA ELISA kits (BD PharMingen, San Diego, USA), according to the manufacturer's recommendations.

### Inhibition-ELISA for detection of *P. brasiliensis* circulating antigen in serum

Inh-ELISA was performed as previously described [21]. Briefly, inhibition standard curve was constructed by adding different concentrations of *P. brasiliensis* gp43 (from 1 ng to 30 µg/ml) in 100 µl of normal serum and then adding 100 µl of the standardized concentration of monoclonal antibody (MAb) anti-gp43 (10 µg/ml). Serum samples (100 µl) were added to 100 µl of MAb anti-gp43. Normal serum was used as a negative control. Polystyrene plates (Corning Costar Co., Corning, USA) were coated with 500 ng of gp43 in 0.06 M carbonate buffer (pH 9.6) per well (100 µl/well) overnight at 4°C. After, the plates were blocked by incubation with 200 µl of 1% bovine serum albumin in PBS per well for 1 h at 37°C; washed 3 times and 100 µl from inhibition standard curve, samples and controls were added per well and allowed to stand for 2 h at 37°C. After being washed 3 times, 100 µl of goat anti-mouse immunoglobulin G-peroxidase (Sigma) was added, and the plates were incubated for 1 h at 37°C. After further washings, the reaction was developed with a solution of o-phenylenediamine (0.5 mg/ml; Sigma) and 0.005% H_2_O_2_. The reaction was stopped with 4 N H_2_SO_4_ after 8 to 10 min of incubation in the dark. Optical densities were measured at 490 nm on a PowerWave X microplate reader (Bio-Tek Instruments, Inc., Winooski, USA). The degree of inhibition in MAb binding was shown to be reciprocal to the concentration of circulating antigen in the sample. The cutoff point was established as the receiver operator characteristic (ROC) curve.

### Statistical analysis

Statistical determinations of the difference between means of experimental groups were performed using two-tailed Mann-Whitney U-test. Differences which provided *P*<0.05 were considered to be statistically significant. All experiments were performed at least three times.

## Results/Discussion

**Figure 1:**
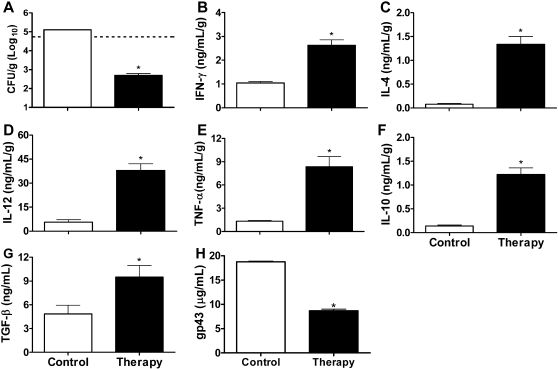
*P. brasiliensis*-infected mice treated with CFA present remarkable decrease of fungal burden and increase of cytokines production. Mice inoculated with 1×10^6^ yeast cells were treated with CFA (therapy) or IFA (control) on day 20 postinfection. The lung homogenates obtained from these mice on day 30 after treatment were analyzed for CFU (A), IFN-γ (B), IL-4 (C), IL-12 (D), TNF-α (E), IL-10 (F), TGF-β (G). In the same period the levels of *P. brasiliensis* circulating antigen was analyzed in serum (H). Dashed line in panel A represents the amount of viable yeasts at the day of treatment (20 days postinfection). Data are reported as the mean±standard deviation for three mice per group performed in duplicate. *P<0.05 compared to control group (two-tailed Mann-Whitney U-test).

**Figure 2:**
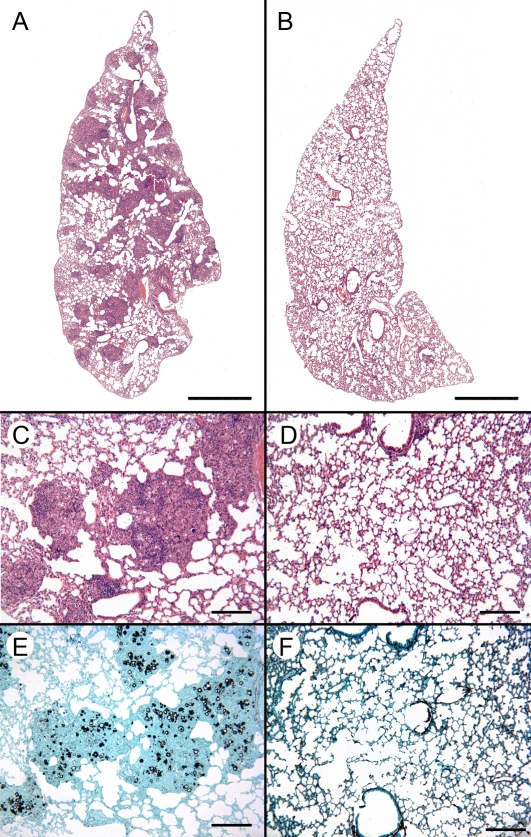
Therapy with CFA leads to resolution of the pulmonary lesions in *P. brasiliensis*-infected mice. *P. brasiliensis*-infected mice were treated with IFA (A, C and E) or CFA (B, D and F) 20 days postinfection. The lung sections obtained at day 30 after treatment were fixed in formalin, paraffin embedded, cut into 5 µm sections, stained with H&E (A–D) or Grocott (E and F), and analyzed by light microscopy. Scale bars on panels A and B indicate 1 mm, on D–G 200 µm.

The depression of cell-mediated immune responses has been associated with severe PCM in humans and in the experimental host [1],[15],[16],[22]. However, the propensity for persistence of the fungus in infected tissues appears to be consequence of cell-mediated immune dysregulation with suppression of Th1 and overexpression of Th2 responses [12]–[14].To evaluate whether therapeutic immunostimulation is able to interfere in experimental murine PCM and restore the host immune response, we selected immunomodulators for therapy strategy based on the induction of Th1 or Th2 immune response. Since CFA supports a Th1 status, while incomplete Freund's adjuvant (IFA) promotes a Th2 status [23], BALB/c mice were divided into two groups and treated with CFA or IFA on day 20 after infection with *P. brasiliensis*. The progression of *P. brasiliensis* infection was determined by lung histopathology and analysis of colony-forming unity (CFU), parameters that are considered trustworthy to discriminate susceptible and resistant mice to systemic fungal infection [9],[12],[19],[24]. At 20 days of infection the mice presented 5.8×10^4^ CFU/g of lung tissue (Figure 1A, dashed line) and compact granulomas (data not shown), for this reason, this time was chosen for the treatment regimens. On day 30 after treatment (50 days postinfection), the lungs from IFA-treated mice presented multiple sites of focal and confluent epithelioid granulomas with lymphomonocytic halos circumscribing a high number of viable and nonviable yeast cells (Figure 2A, C and E). Morphometric analysis of the lungs from IFA-treated mice revealed a number of granulomas of 41±5.2, with a relative area of 40.7±6.2%. These granulomas presented 12.2±1.8% of yeast cells and 6±0.6% collagen (data not shown). In contrast, in the *P. brasiliensis*-infected mice treated with CFA, no granulomas or yeast cells were seen in the pulmonary sections examined and a well-preserved alveolar architecture was observed on day 30 after treatment (Figure 2B, D and F). Most importantly, the treatment with CFA induced a 2.4 log reduction in the fungal burden when compared to the IFA-treated mice, corresponding to 99% less CFU (Figure 1A). The CFU data are in agreement with the histopathology analyses, pointing out that therapeutic immunostimulation led to an increased clearance of fungal burden from lungs.

In order to evaluate the impact of the treatment with adjuvant, the animals were weighed weekly until the study end point. We observe that the animals of therapy group gained more weight (20%) than the control group (data not shown). These results can be correlated with a good prognostic in the PCM.

When we analyzed the production of pro and anti-inflammatory cytokines in the supernatants of lung homogenates from the *P. brasiliensis*-infected BALB/c mice treated with CFA or IFA, we observed that the IFA-treated group produced low levels of IFN-γ, IL-4, IL-12, TNF-α, IL-10 and TGF-β (Figure 1B–G), suggesting a suppression of the immune response in these animals. In contrast, CFA-treated mice produced high levels of these pro and anti-inflammatory cytokines (Figure 1B–G). Although many reports have demonstrated that the Th2 pattern is associated with a severe disease, whereas a Th1-biased immune response is linked to the asymptomatic and mild forms of PCM [9]–[14], others have shown that the induction of inflammatory cytokines, such as IFN-γ and TNF-α, can lead to overproduction of nitric oxide that has been associated with suppression of cell immunity [25]–[27]. Recently, it was demonstrated that the anti-inflammatory Th2 cytokine IL-4 has a dual role in PCM, leading to a protective or a disease-promoting effect depending on the genetic background of the host [28]. Regarding TGF-β, we observed that this cytokine is produced by pulmonary epithelium, so we hypothesized that it might contribute to the lung tissue renewal (unpublished data). In this study we obtained an effective protection against *P. brasiliensis* infection even in the presence of anti-inflammatory cytokines, suggesting that, in this therapy model, the protective effect against PCM seems to be dependent on the induction of a mixed Th1/Th2 immune response pattern. The production of both inflammatory and anti-inflammatory cytokines is extremely helpful to balance the immune response, since anti-inflammatory cytokines can control the inflammatory responses, which can result in local pathology and systemic and centrally controlled adverse events. CD4^+^ T cells also play a role in the regulation of inflammation [29]. On the basis of the differences between the groups treated with CFA or IFA, we suggest that the protection induced by CFA injection was due to a noticeable increase in the pulmonary levels of cytokines, which probably broke the immunosuppression status observed in the infected mice treated with IFA. Nonetheless, we cannot exclude the involvement of other mechanisms, such as modulation by regulatory T cells [30], apoptosis in the antigen-specific T cells [31], and Fas-FasL and CTLA-4 expression [32].

The levels of circulating antigen in the mice infected and treated with IFA were two-fold higher than those treated with CFA (Figure 1H). These results supported by other reports that showed that the depression of cell-mediated immunity is associated with the high levels of specific circulating antibodies or soluble antigens in disseminated disease [13],[15],[21].

Although many studies on protection against PCM have been performed, only few of them have reported the efficacy of the immunostimulatory therapy. In one of these studies, the therapy with peptide p10 from gp43, emulsified in CFA, and chemotherapy was used in an attempt to improve the treatment of PCM [33]. The combined treatment showed a beneficial effect when administered at 48 h or 30 days after challenge. However, the control mice that received only CFA and the non-immunized mice presented similar lung fungal burden. These data are in contrast to those observed herein. This difference might be due to distinct experimental protocols used, such as challenge route, dose, and treatment regimen. Nevertheless, other reports have demonstrated that the use of immunostimulatory therapy can lead to a positive prognostic in fungal diseases [34]–[36]. Basically, therapeutic immunostimulation can be used by reinforcing or broadening defenses when specific immune responses are unable to do this during the natural course of the PCM.

The present study demonstrated that a single-dose administration of the Th1-inducing adjuvant (CFA) in *P. brasiliensis*-infected mice was sufficient to break the anergy observed in these animals restoring their ability to mount an effective immune response to the fungus. While the control mice presented large amount of yeasts and extensive sites of parenchymal lung injury, the CFA-treated mice were capable to control not only the fungal systemic dissemination but also its growth, leading to a noticeable fungal clearance without apparent lung injury. Our results indicate that Th1-inducing adjuvant proved to be a valuable tool in the treatment of PCM. Overall, these data open new possibilities for the potential use of Th1-inducing adjuvant not only as a sole therapy but also as an adjunct to conventional antifungal therapy against PCM, improving the regular chemotherapy and reducing the time of treatment.
